# Unraveling pathophysiologic mechanisms contributing to symptoms in patients with post‐acute sequelae of COVID‐19 (PASC): A retrospective study

**DOI:** 10.14814/phy2.15754

**Published:** 2023-06-21

**Authors:** Wendel Dierckx, Wilfried De Backer, Kris Ides, Yinka De Meyer, Eline Lauwers, Erik Franck, Jan De Backer

**Affiliations:** ^1^ Centre for Research and Innovation in Care, Faculty of Medicine and Health Sciences University of Antwerp Antwerp Belgium; ^2^ Multidisciplinary Medical Center Kontich Belgium; ^3^ Faculty of Medicine and Health Sciences University of Antwerp Antwerp Belgium; ^4^ Laboratory of Experimental Medicine and Pediatrics, Faculty of Medicine and Health Sciences University of Antwerp Antwerp Belgium; ^5^ FLUIDDA NV Kontich Belgium; ^6^ CoSys Research Lab, Faculty of Applied Engineering University of Antwerp Antwerp Belgium; ^7^ Flanders Make Strategic Research Center Lommel Belgium; ^8^ Department of Pediatrics Antwerp University Hospital Edegem Belgium; ^9^ Clinical Operations FLUIDDA NV Kontich Belgium; ^10^ FLUIDDA Inc. New York City New York USA

**Keywords:** COVID‐19, functional respiratory imaging, PASC

## Abstract

Patients with post‐acute sequelae of COVID‐19 (PASC) present with a decrease in physical fitness. The aim of this paper is to reveal the relations between the remaining symptoms, blood volume distribution, exercise tolerance, static and dynamic lung volumes, and overall functioning. Patients with PASC were retrospectively studied. Pulmonary function tests (PFT), 6‐minute walk test (6MWT), and cardiopulmonary exercise test were performed. Chest CT was taken and quantified. Patients were divided into two groups: minor functional limitations (MFL) and severe functional limitations (SFL) based on the completed Post‐COVID‐19 Functional Status scale (PCFS). Twenty one patients (3 M; 18 FM), mean age 44 (IQR 21) were studied. Eighteen completed the PCFS (8 MFL; 10 SFL). VO_2_max was suboptimal in both groups (not significant). 6MWT was significantly higher in MFL‐group (*p* = 0.043). Subjects with SFL, had significant lower TLC (*p* = 0.029). The MFL‐group had more air trapping (*p* = 0.036). Throughout the sample, air trapping correlated significantly with residual volume (RV) in L (*p* < 0.001). An increase in air trapping was related to an increase in BV5 (*p* < 0.001). Mean BV5 was 65% (IQR 5%). BV5% in patients with PASC was higher than in patients with acute COVID‐19 infection. This increase in BV5% in patients with PASC is thought to be driven by the air trapping in the lobes. This study reveals that symptoms are more driven by occlusion of the small airways. Patients with more physical complaints have significantly lower TLC. All subjects encounter physical limitations as indicated by suboptimal VO_2_max. Treatment should focus on opening or re‐opening of small airways by recruiting alveoli.

## 1 INTRODUCTION

In December 2019, the SARS‐CoV‐2 emerged and caused a widespread pandemic. Common signs and symptoms were fever, cough, and tiredness. While many patients were asymptomatic or experienced moderate symptoms, 15% of the patients evolved towards a more severe disease and requiring hospitalization (Michelen et al., 2021).

In a substantial number of patients, symptoms remain beyond the acute phase of the infection. These post‐acute sequelae of COVID‐19 (PASC), commonly referred to as long COVID, affect COVID‐19 survivors at all levels of disease severity, even younger adults, children, and those not hospitalized (Yong, 2021). The definition of PASC is evolving and can be divided into two categories. Symptoms and signs persisting 4–12 weeks after the acute infection are labeled as subacute symptoms. Remaining sequelae 12 weeks after the initial onset are described as chronic PASC. This chronic condition can be very heterogeneous (Datta et al., 2020). Over 100 persistent symptoms of long COVID are summarized in systematic reviews. The most common symptoms are fatigue and dyspnea however also cognitive impairment (brain fog, memory loss) and chest or joint pain are frequently reported (Hayes et al., 2021; Lopez‐Leon et al., 2021).

The burden of these and other long‐lasting symptoms lead to a decrease in physical fitness.

In hospitalized patients with acute severe COVID‐19 infection, changes in pulmonary vascular volume distribution as measured by quantitative CT analysis have been observed. Potentially due to micro emboli and vascular remodeling, there was a shift in blood distribution from smaller to larger vessels (Lins et al., 2020). These changes can be partially responsible for the severe hypoxemia (Dierckx et al., 2022) and the subsequent need for invasive ventilation. A low blood volume in pulmonary vessels smaller than 5 mm^2^ (BV5%) in cross‐sectional area, was associated with higher need for intubation and mortality in patients with acute COVID‐19 (Morris et al., 2021).

The contribution of this latter phenomenon to the persistent exercise intolerance and dyspnea in patients with PASC has yet to be determined. The aim of this paper is to describe the pulmonary blood volume distribution in PASC patients. In addition, the purpose is to unravel the pathophysiologic mechanisms contributing to symptoms in patients with PASC. This will lead to a better understanding of the relation between the remaining symptoms, blood volume distribution, exercise tolerance, static and dynamic lung volumes, and overall functioning.

## 2 MATERIALS AND METHODS

### 2.1 Study design

A retrospective observational study was conducted in a second line multidisciplinary medical practice MedImprove located in Kontich (Flanders, Belgium).

### 2.2 Participants

#### 2.2.1 Inclusion and exclusion criteria

Data of patients at MedImprove with persisting respiratory symptoms at least 4 weeks after the initial infection with COVID‐19 were collected between August 2021 and October 2022. All male and female patients >18 years were enrolled (convenience sample). Only subjects with symptoms of comorbidities before COVID‐19 infection, such as myalgic encephalomyelitis/chronic fatigue syndrome (ME/CFS) or previous heart or lung diseases, that might interfere with the symptoms of long covid were excluded.

### 2.3 Measurements and endpoints

Demographic data were collected including age, gender, height, and weight. After a general clinical examination, assessment of current symptoms and concomitant medication, baseline measurements were executed.

All patients performed a pulmonary function test (PFT) (Vyntus Body, Vyaire Medical Chicago). Dynamic lung volumes as expiratory capacity in 1 s (FEV1) and forced vital capacity (FVC) were measured by spirometry. Static lung volumes as total lung capacity (TLC) and functional residual capacity (FRC) were measured by body plethysmography. The diffusing capacity of the lung for carbon monoxide (DLCO) was measured by a 10 s breath hold maneuver at TLC, after inhalation of the diffusion gas O_2_ (20.9%), CO (0.3%), CH_4_ (0.3%), and N (78.5%). To evaluate the carbon monoxide diffusion constant, the DLCO was divided by the alveolar volume (VA).

Non‐contrast, thin‐slice, volumetric chest CT was acquired at TLC and functional residual capacity (FRC). 3D reconstructions of the lungs and pulmonary vasculature were created. An automated blood vessel segmentation algorithm performs an eigenvalue analysis of the Hessian matrix to enhance and identify tubular structures, by returning the probability of each voxel belonging to tubular structure based on shape analysis (Yang et al., 2014). Next, Hounsfield unit (HU) thresholds are used to limit the vessels. The HU thresholds are based on the vessels size and are defined by an automated adaptive iterative threshold method. In the preprocessing, a gradient anisotropic diffusion filter is applied, and a region of interest is defined to remove some false positives. Subsequently, the smaller non‐connected parts are removed. To account for the effects of slice thickness on results, sensitivity analysis was performed. Volumes were then computed from the cross‐sectional area of each vessel. Following the convention established by Rahagi et al., the volume of blood contained in vessels below 5 mm^2^ cross sectional area (down to a cutoff of 1.25 mm^2^) was termed “BV5”. We additionally defined BV5‐10 as the volume of blood contained in vessels with cross‐sectional area between 5 and 10 mm^2^, and BV10 as the volume contained in vessels with cross‐sectional area above 10 mm^2^. We refer collectively to these quantities as BVX. To account for variation in lung volume, we chose to normalize BVX by total pulmonary blood volume. This permits for the computation of a “BV spectrum”, a curve representing the percent of total pulmonary blood volume contained within vessels of a given caliber as a function of cross‐sectional area. It had previously been observed in analysis of scans from healthy volunteers that this yielded values with very low variance over all scales.

Functional respiratory imaging (FRI)‐based air trapping is defined as all the intrapulmonary voxels with HUs between −1024 and −850 using the expiratory scans at FRC. A Binominal Blur filter is applied and a mask within these thresholds is created, being intersected with each lobe mask to make the regional air trapping masks. The 3D models are then created from these masks.

By identifying and grouping the voxels that represent the air in the lungs, the lung volume (L) can be determined from the scans. During segmentation, lung lobes are separated by identifying the fissure planes on the CT images and using these surfaces as cutting objects. This means that not only the total lung volume is determined, but also the volume of each lobe individually.

Cardiorespiratory fitness (VO_2_ peak in mL/kg/min) was measured by a maximal incremental cardiopulmonary exercise test using Vyntus CPX device (Vyaire Medical Chicago) and an ergocycle (GE Vivid S60 N NOR v204) with monitoring of ECG (Cardiosoft GE). After a 3 min warm up, the protocol started with 5, 10, or 20 W, followed by an increasing of the workload with 5–15 Watt per minute, depending on the patient's baseline condition. The patient was asked to cycle with a cadence of 60 rotations per minute. This protocol was followed until exhaustion.

Other assessments performed were the 6‐minute walk test (6MWT), Borg Dyspnea Scale (Borg) and the Post‐COVID‐19 Functional Status scale (PCFS) (Klok et al., 2020). The PCFS is a global instrument that correlates with the quality of life, dyspnea and mental health and is also a suitable instrument to screen for patients requiring careful follow‐up after COVID‐19 infection (Benkalfate et al., 2022). It categorizes patients into four groups from neglectable (Grade 1) to severe (Grade 4) functional limitations. Given the small number of patients in this cohort, these PCFS classes were merged into two groups: Grades 1 and 2 were considered minor functional limitations (MFL), and Grades 3 and 4 were considered severe functional limitations (SFL).

### 2.4 Statistics

SPSS Statistics 28.0.1.1 (IBM) was used for analysis. All analyses were evaluated using a significance cutoff of *p* < 0.05.

Demographic data were examined descriptively to gain an understanding of the participating population. Discontinuous variables were described using frequency distributions. Variables were described using mean and SD (parametric), or median and interquartile range (nonparametric). Mann–Whitney *U* was used to analyze the difference between groups and Spearman's rho for correlations.

## 3 RESULTS

### 3.1 Basic characteristics

Between August 2021 and October 2022, a total of 21 subjects (3 male; 18 female), mean age 44 (SD 13) years, presented at the clinical practice of MedImprove with persisting respiratory symptoms at least 4 weeks after the initial COVID‐19 infection. Previous to the latter, these 21 patients did not have any comorbidities, respiratory disease, nor issues (ME/CFS), and were all enrolled in the retrospective study. None of the patients were hospitalized during their acute COVID‐19 infection. All subjects were initially treated symptomatically with paracetamol and rest. Only one subject was administered oral prednisolone. None of the cohort was on inhaled therapy previously to their first consultation. Descriptive data of the basic characteristics are given in Table 1.

**TABLE 1:** Descriptive statistics of basic characteristics.

	*N*	Mean	Median	Range	IQR 25–75th
Age (years)	21	44	40	49	33–54
Gender: M/F/X	21	3 M	18 F	0 X	—
Height (cm)	21	170	172	29	165–175
Weight (kg)	21	78	75	73	68–92
BMI (kg/m^2^)	21	27	26	21	22–31
Time positive PCR—first consultation (days)	21	158	156	393	40–260

The results of the PFTs, 6 MWT, and ergospirometry are given in Table 2.

**TABLE 2:** Descriptive statistics of tests.

	*N*	Mean	Median	Range	IQR 25–75th
Forced expiratory volume in 1 s (FEV1) (%)	21	92	93	70	81–101
Forced vital capacity (FVC) (%)	21	96	97	63	89–99
Tiffeneau index (FEV1/FVC) (%)	21	77	79	27	70–83
Residual volume (RV) (%)	21	95	93	87	80–111
Total lung capacity (TLC) (%)	21	95	102	43	86–105
DLCO (%)	21	94	93	62	86–102
Diffusing capacity (DLCO/VA) (%)	21	105	106	88	91–120
Specific airway resistance (sRaw) (%)	21	124	128	99	98–147
6 Minute walk distance (6MWD) (m)	19	481	497	640	397–618
6 Minute walk distance (6MWD) % predicted (%)	19	65	67	87	59–75
VO_2_ max predicted (%)	21	79	83	74	59–93
O_2_ pulse at peak load (mL)	21	11	11	7	9–12
O_2_ pulse at peak load % predicted (%)	21	100	96	82	87–114
EqO_2_ at peak load	21	30	30	22	26–32
EqCO_2_ at peak load	21	28	28	13	24–29
Ergospirometry peak load (W)	21	104	100	160	65–140

The median of the baseline Borg Dyspnea score of 18 subjects was 2, with a range of 7 (IQR 2).

Eighteen subjects completed the PCFS. With eight subjects experiencing mild functional limitations (MFL) and 10 subjects with SFL with no differences in age (*p* = 0.657), BMI (*p* = 0.23) or Borg Dyspnea Score (*p* = 0.693) between these groups.

### 3.2 Exercise capacity

Throughout the whole sample, there are large ranges in 6MWD (640 m) and ergospirometry peak load (160 W).

**FIGURE 1:**
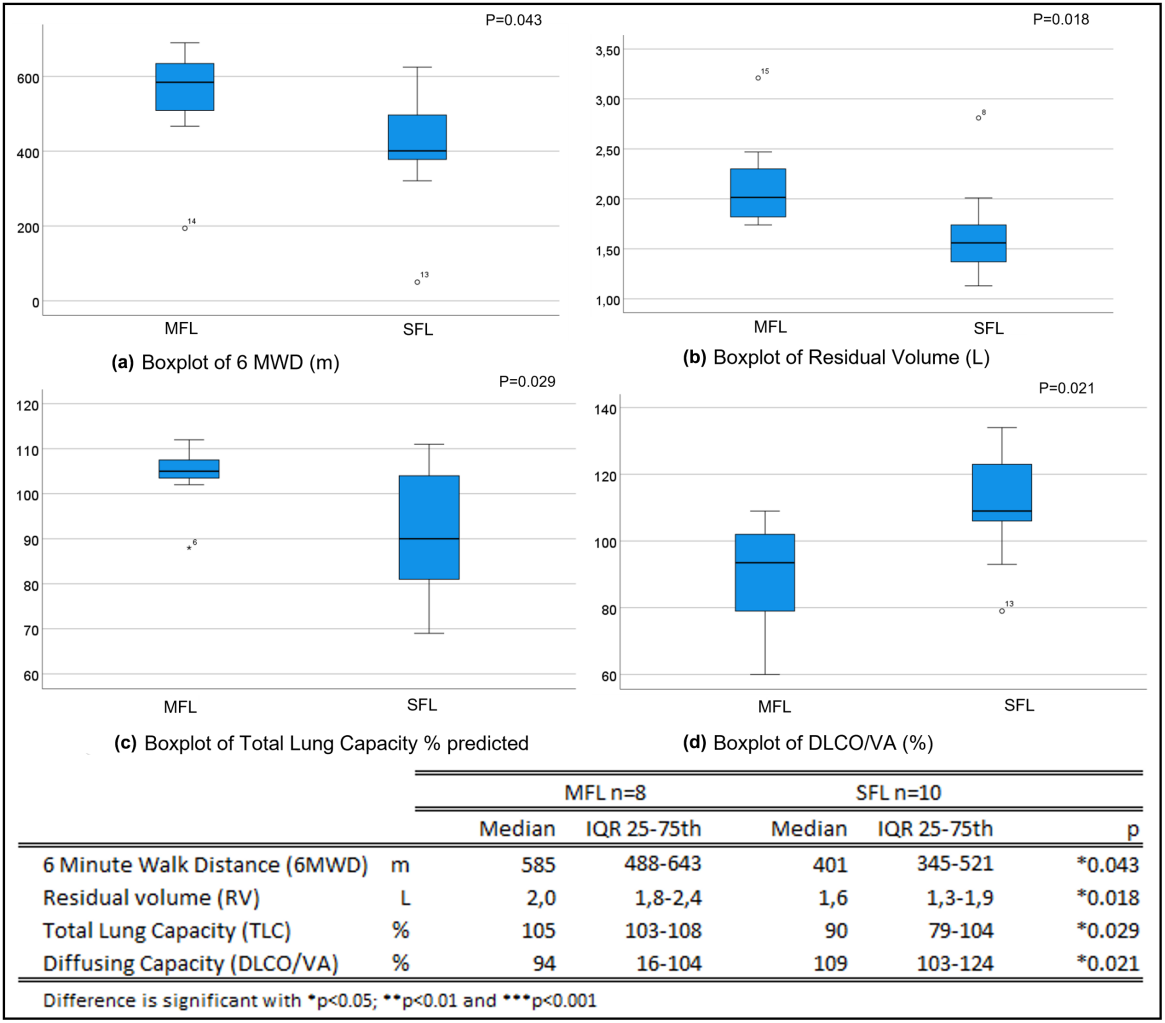
Boxplots of differences between mild functional limitations (MFL) and severe functional limitations (SFL) groups, for (a) 6 MWD; (b) RV; (c) TLC and (d) DLCO/VA.

A significant difference in 6MWD (*p* = 0.043) between the MFL and SFL group was found reflecting the severity of the functional impairments in the two groups (Figure 1a).

The overall functioning of all patients was low when considering the ergospirometry results. The group with MFL reached a mean VO_2_max predicted of 87.63% (SD 21.83%) and the SFL‐group reached a mean VO_2_max predicted of 74.40% (SD 19.43%). Between the two groups, although numerically different, no significant difference was found in the maximal exercise tolerance (*p* = 0.13). Even no significant differences were found between the two groups in O_2_pulse (*p* = 0.72), EqO_2_ at peak (*p* = 0.42), nor EqCO_2_ at peak (*p* = 0.66).

### 3.3 Pulmonary function test

Lung function parameters uncovered large differences between patients with wide ranges in FEV1, RV, TLC, and DLCO/VA. When comparing the MFL and SFL group, significant differences were found in RV Liter (*p* = 0.018; Figure 1b), TLC predicted (*p* = 0.029) (Figure 1c), and DLCO/VA (*p* = 0.021; Figure 1d). No evidence of differences were found in FVC predicted (102.75% vs 91.70%, *p* = 0.091), FEV1 predicted (94.13% vs 89.70%, *p* = 0.560), and DLCO (85.75% vs 96.80%, *p* = 0.056).

### 3.4 Chest CT

Scans were evaluated by trained radiologists. Visually, all subjects had a normal CT‐thorax scan. There were no signs of ongoing parenchymal infiltrates or fibrotic tissue.

Table 3 shows the descriptive statistics of the FRI.

**TABLE 3:** Descriptive statistics functional respiratory imaging.

	*N*	Mean	Median	Range	IQR 25–75th
BV5 (%)	21	65	65	16	62–67
BV 5‐10 (%)	21	18	18	7	16–20
BV 10 (%)	21	17	17	11	16–19
Total blood vessel volume (mL)	21	140	140	77	121–161
Total air trapping (%)	17	19	10	56	3–37

Abbreviations: BV5, vessels less than 5 mm^2^ in cross sectional area; BV 5‐10, vessels bigger than 5 mm^2^ and less than 10 mm^2^ in cross sectional area; BV 10, vessels bigger than 10 mm^2^ in cross sectional area.

**FIGURE 2:**
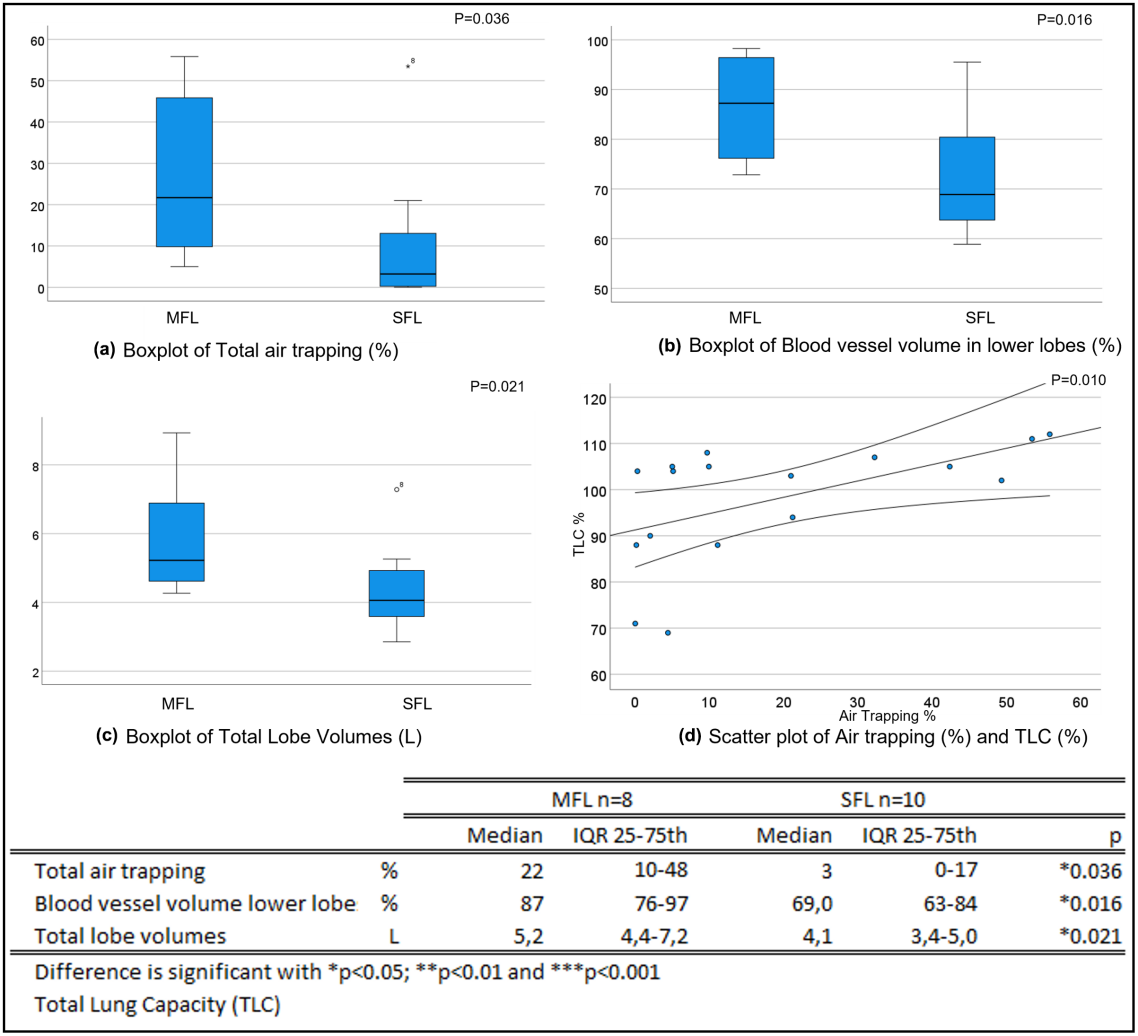
Boxplots of differences between mild functional limitations (MFL) and severe functional limitations (SFL) groups, for (a) Total air trapping; (b) Blood vessel volume in lower lobes; (c) Total lobe volumes and (d) scatter plot of Air trapping and TLC.

The quantified CT scans revealed significant differences between the two groups for several parameters, including air trapping (*p* = 0.036; Figure 2a), image‐based blood vessel volume in the lower lobes (*p* = 0.016; Figure 2b), and image‐based lobe volume (*p* = 0.016; Figure 2c).

Throughout the sample, air trapping correlated significantly with RV in L (*p* < 0.001), RV predicted (*p* = 0.012) and TLC % (*r* = 0.61; *p* = 0.010; Figure 2d). No other significant correlation was found between 6 MWD, VO_2_ max, or peak load and quantitative CT thorax parameters. Only oxygen pulse at peak load in % predicted, correlated significant with BV5‐10 (*r* = −0.51; *p* = 0.020) and BV5 (*r* = 0.44; *p* = 0.047). The mixed model considering BV5 and air trapping on a lobar level, showed that an increase in air trapping was related to an increase in BV5 (*p* < 0.001).

## 4 DISCUSSION

Twenty one patients were studied with persistent symptoms at least 4 weeks after an acute infection with COVID‐19. None of the patients did have any comorbidities, respiratory disease, nor issues of ME/CFS prior to their acute COVID‐19 infection. For the latter, they were treated symptomatically at home. Of the 18 subjects that completed the PCFS questionnaire, eight subjects were in the group with MFL and 10 of them had SFL.

The significant difference in the 6MWD reflects the impairment of the group with higher functional limitations. The predicted VO_2_ max was higher in the group with MFL, however not significantly different compared to the predicted VO_2_ max in the SFL group. An explanation might be that subjects with MFL, experience more functional limitations during heavy physical exertion than during mild exercise.

The pulmonary function testing revealed large ranges in TLC. In the systematic review of Torres‐Castro et al. there was a main finding of diminished TLC (Torres‐Castro et al., 2021). The Mann–Whitney U between the two PCFS groups in this study, revealed that a higher TLC correlates with less functional limitations. Patients with lower TLC tend to have more functional limitations. This might explain the systematic reviews' findings of low TLC, as their study population were all hospitalized patients, measured 6 weeks after discharge.

The air trapping can have many causes (Miller et al., 2014), as patients did not have interstitial lung disease or bronchiectasis before their COVID‐19 infection. The current CT thorax scans did not reveal any sign of ongoing parenchymal infiltrates or fibrotic tissue. It is more likely that there was a chronic bronchitis or unspecified small airways disease, which resulted in partially occluded airways. Air trapping during the acute COVID‐19 infection was already reported (Dai et al., 2020). In quantified CT scans 6 months after hospital discharge for COVID‐19 infection, 29% of the scans revealed air trapping (Jia et al., 2022). When considering the significant difference in air trapping between the two PCFS‐groups, we can conclude that the air trapping is not the driving parameter for the physical limitations. The patients with more air trapping, showed higher lobe volumes measured by quantified CT scan and higher total lung capacity measured by PFT. Compared to the SFL group, they presented with a higher RV.

The DLCO/VA was significant lower in the MFL group (*p* = 0.021). In the SFL group, a decreased TLC was observed what might lead to a higher proportion in DLCO/VA and might explain the significant difference between both groups. The BV5 correlated significantly with air trapping. The more air trapping, the higher the BV5. The expansion of the lobes due to the air trapping can cause compression on the (not quantified) intra‐alveolar blood vessels with a shift of blood flow to the blood vessels with a diameter of 5 mm^2^. It is also known that endothelitis (Cavallieri et al., 2022) in the capilary vessels of the lungs can cause this shift in blood flow to the larger vessels. Alveolar–capillary damage or microvascular pathology might result in a declined DLCO/VA (Hughes & Pride, 2012).

However, a total occlusion of the small airways resulting in micro‐atelectasis and shunting, will cause more SFL and contributes to the decrease in total lung capacity. The DLCO/VA was significantly higher in patients with SFL, which might indicate that they have no impairment in their diffusing capacity. These patients with SFL have less lobe volume resulting in significantly less blood volume in the lower lobes compared to patients with MFL (*p* = 0.016).

The overall BV5% of all patients in this study with PASC is 64.62% (SD 3.99%) and therefore not reduced as it was seen in patients with acute Covid infection where a BV5% of 35% was measured (Lins et al., 2020).

The treatment of these patients with PASC should focus on the opening or re‐opening of the small airways by recruting alveoli. Therefore, a combination of physical activity and if needed medical treatment (ICS, bronchodilatation) is needed.

This study has several limitations. All data was retrospectively collected in one center. All included subjects had chosen to present to a clinic with post‐COVID symptoms which might introduce unmeasured confounders. The sample size used in this proof of concept study is discrete and needs to be expanded for further validation and to phenotype these patients more in detail. Most patients were suspected of being infected with the Omicron variant, however this was not always confirmed and therefore not included in the manuscript. In this study no blood parameters were included. Blood analysis could be used in additional research to verify the findings. The quantified CT has no reference values but can be used for intra patient comparisons and for this population, the baseline measurement will be used to evaluate the impact of pulmonary rehabilitation in future research.

Additional research needs to be focused on the impact of pulmonary rehabilitation and the evolution of the clinical outcomes of those patients.

## 5 CONCLUSIONS

The BV5% in patients with PASC was higher than measured in patients with acute COVID‐19 infection. This increase in BV5% in patients with PASC is thought to be driven by the air trapping in the lobes. However, this study reveals that symptoms are more driven by occlusion of the small airways. Patients with more physical complaints have a significantly lower TLC compared to patients with less physical complaints, but also patients with higher TLC encounter physical limitations as indicated by suboptimal VO_2_max. The treatment should focus on the opening or re‐opening of the small airways by recruiting alveoli. This is work for a multidisciplinary team of pulmonologists, physiotherapists and nurses, to provide pulmonary rehabilitation including exercise training, medical treatment if needed, and a follow‐up of the pulmonary function parameters, especially TLC.

## AUTHOR CONTRIBUTIONS

Wendel Dierckx—conceptualization, data curation, formal analysis, investigation, methodology, project administration, software, supervision, validation, visualisation, writing original draft, writing review and editing. Wilfried De Backer—conceptualization, data curation, formal analysis, investigation, methodology, software, supervision, validation, visualisation, writing original draft, writing review and editing. Kris Ides—conceptualization, data curation, formal analysis, methodology, project administration, supervision, validation, visualisation, writing original draft, writing review and editing. Yinka De Meyer—data curation, project administration, writing review and editing. Eline Lauwers—formal analysis, writing review and editing. Erik Franck—conceptualization, writing review and editing. Jan De Backer—software, visualisation, writing review and editing.

## ETHICS STATEMENT

All subjects in this practice signed an informed consent approving that their medical data can be pseudonymized utilized for research purposes. This monocenter study was reviewed and ethically approved by the Ethical Committee of the University Hospital of Antwerp on December 12, 2022 (Project ID 5050).
